# Inactivation of Cyclin-Dependent Kinase 5 in Hair Cells Causes Hearing Loss in Mice

**DOI:** 10.3389/fnmol.2018.00461

**Published:** 2018-12-11

**Authors:** Xiaoyan Zhai, Chengcheng Liu, Bin Zhao, Yanfei Wang, Zhigang Xu

**Affiliations:** ^1^Shandong Provincial Key Laboratory of Animal Cells and Developmental Biology, School of Life Sciences, Shandong University, Qingdao, China; ^2^Department of Otolaryngology-Head and Neck Surgery, The Second Hospital of Shandong University, Jinan, China; ^3^Shenzhen Research Institute of Shandong University, Shenzhen, China; ^4^Shandong Provincial Collaborative Innovation Center of Cell Biology, Shandong Normal University, Jinan, China

**Keywords:** inner ear, hair cells, stereocilia, hearing loss, CDK5

## Abstract

Cyclin-dependent kinase 5 (CDK5) is abundantly expressed in post-mitotic cells including neurons. It is involved in multiple cellular events, such as cytoskeletal dynamics, signaling cascades, gene expression, and cell survival, et al. Dysfunction of CDK5 has been associated with a number of neurological disorders. Here we show that CDK5 is expressed in mouse cochlear hair cells, and CDK5 inactivation in hair cells causes hearing loss in mice. CDK5 inactivation has no effect on stereocilia development in the cochlear hair cells. However, it affects stereocilia maintenance, resulting in stereocilia disorganization and eventually stereocilia loss. Consistently, hair cell loss was significantly elevated by CDK5 inactivation. Despite that CDK5 has been shown to play important roles in synapse development and/or function, CDK5 inactivation does not affect the formation of ribbon synapses of cochlear hair cells. Further investigation showed that CDK5 inactivation causes reduced phosphorylation of ERM (ezrin, radixin, and moesin) proteins, which might contribute to the stereocilia deficits. Taken together, our data suggest that CDK5 plays pivotal roles in auditory hair cells, and CDK5 inactivation causes hearing loss in mice.

## Introduction

In the mammalian cochlea, hair cells are arranged into one row of inner hair cells (IHCs) and three rows of outer hair cells (OHCs) that are interlaced with various types of supporting cells (Schwander et al., 2010). As the auditory receptor cells, hair cells are characterized by their hairy-looking, F-actin-based stereocilia on the apical surface. For each hair cell, dozens to hundreds of stereocilia are organized into several rows of increasing heights, forming a staircase-like pattern. The mechanoelectrical transduction (MET) channels have been suggested to localize at the tips of shorter stereocilia (Beurg et al., 2009). Stereocilia are organized in a flattened U-shaped (IHCs) or V-shaped (OHCs) pattern, while the tallest stereocilia are positioned close to the vertex. The vertices of stereocilia in all hair cells point away from the center of the cochlea, establishing the planar cell polarity (PCP) of the cochlear epithelia. A single microtubule-based kinocilium localizes at the vertex of stereocilia, but degenerates at late developmental stage in cochlear hair cells, implying that it is not necessary for MET (Lindeman et al., 1971). Instead, kinocilium was believed to play pivotal roles in stereocilia development and cochlear PCP establishment (Jones et al., 2008). Stereocilia and kinocilium together constitute the so-called hair bundle. The detailed mechanisms of hair bundle development and maintenance as well as hair cell PCP establishment still remain elusive.

Cyclin-dependent kinase 5 (CDK5) is a proline-directed serine/threonine kinase. It is abundantly expressed in post-mitotic cells including neurons and is necessary for cell differentiation (Tsai et al., 1993; Nikolic et al., 1996). It has been shown that CDK5 plays important roles in multiple cellular events, such as cytoskeletal dynamics, signaling cascades, gene expression, and cell survival, et al (Su and Tsai, 2011). Similar to other CDKs whose kinase activity needs to be activated by cyclins, CDK5 is activated by specific activators, including p35, p39, cyclin I (CCNI), and cyclin I-like (CCNI2) (Tsai et al., 1994; Tang et al., 1995; Brinkkoetter et al., 2009; Liu et al., 2017).

Dysfunction of CDK5 has been associated with a number of neurological disorders including Alzheimer’s disease, amyotrophic lateral sclerosis (ALS), and Niemann-Pick type C disease (Su and Tsai, 2011). For example, neurotoxic signals such as β-amyloid (Aβ), excitotoxicity, ischemia, and oxidative stress cause cleavage of p35 into p25 and p10 through activating calpain. The resultant p25 causes constitutive activation and mislocalization of CDK5, which eventually leads to neuronal death (Patrick et al., 1999; Lee et al., 2000). Germline inactivation of *Cdk5* gene in mice abolishes cortical laminar structure and cerebellar foliation, and causes abnormal motor axons and neuromuscular synapses, which eventually leads to perinatal mortality and hampers further examination of the physiological function of CDK5 (Ohshima et al., 1996; Fu et al., 2005). To circumvent this obstacle, tissue-specific inactivation of CDK5 has been employed to study the physiological role of CDK5 in different cells such as certain neurons, hippocampus, and adipose (Hirasawa et al., 2004; Hawasli et al., 2007; Guan et al., 2011; Banks et al., 2015).

CDK5 has been shown to be expressed in chicken auditory hair cells and regulate the membrane expression and kinetics of BK channel Slo (Bai et al., 2012). Moreover, inhibition of CDK5 with roscovitine induced differentiation of supernumerary hair cells and supporting cells in the developing rat cochlear explant cultures (Malgrange et al., 2003). These results suggested that CDK5 might play an important role in auditory hair cell differentiation and/or function. In order to investigate the physiological role of CDK5 in hearing, we made use of conditional knockout mice that selectively disrupt *Cdk5* gene expression in the hair cells. Our results showed that *Cdk5* inactivation causes hair cell loss and leads to deafness in mice.

## Materials and Methods

### Mice

*Cdk5^lox/+^* mice (Samuels et al., 2007), *EIIa^Cre/+^* (Lakso et al., 1996), and *Atoh1^Cre/+^* (Yang et al., 2010) mice were maintained on a mixed genetic background and genotyped as described previously. *EIIa^Cre/+^*;*Cdk5^lox/lox^* mice (“*Cdk5* ko mice”) die perinatally, therefore *Atoh1^Cre/+^*;*Cdk5^lox/lox^* mice (“*Cdk5* cko mice”) were used in the present work. *Cdk5^lox/lox^* mice (“*Cdk5* wt mice”) were included as control. Whole-mount immunostaining (see below) showed that CDK5 is expressed in *Cdk5^lox/lox^* auditory hair cells, but absent in *Atoh1^Cre/+^*;*Cdk5^lox/lox^* auditory hair cells, confirming successful CDK5 inactivation in hair cells of *Atoh1^Cre/+^*;*Cdk5^lox/lox^* mice.

### Whole-Mount Immunostaining

All steps were performed at room temperature unless otherwise indicated. Dissected organ of Corti explants were fixed with 4% paraformaldehyde (PFA) in PBS for 30 min, followed by permeabilization and blocking with PBT1 (0.1% Triton X-100, 1% BSA, and 5% heat-inactivated goat serum in PBS, pH 7.3) for 30 min. Samples were then incubated with rabbit anti-CDK5 antibody (Santa Cruz, Cat. No. sc-173, 1:100 diluted) or rabbit anti-MYO6 (Cell Signaling Technology, Cat. No. 9200, 1:50 diluted) in PBT1 overnight at 4°C, followed by incubation with Alexa Fluor^®^ 488 donkey anti-rabbit secondary antibody (Invitrogen, Cat. No. A21206, 1:200 diluted) in PBT2 (0.1% Triton X-100 and 0.1% BSA in PBS) for 1 h. After that, samples were incubated with TRITC-conjugated phalloidin (Sigma-Aldrich, Cat. No. P1951) in PBS for 30 min, then mounted in PBS/glycerol (1:1) and imaged with a confocal microscope (LSM 700, Zeiss, Germany). For CtBP2 staining, samples were incubated with mouse anti-CtBP2 antibody (BD, Cat. No. 612044, 1:100 diluted) in PBT1 overnight at 4°C, followed by incubation with Alexa Fluor^®^ 568 goat anti-mouse IgG1 (Invitrogen, Cat. No. A21124, 1:200 diluted) in PBT2 for 1 h and DAPI (Gene View Scientific Inc.) in PBS for 1 h.

### Cryosection Immunostaining

Mouse cochleae were embedded in OCT compound and sectioned in 8–10 μm thickness. After fixation with 4% PFA in PBS for 30 min, samples were permeabilized and blocked with PBT1 for 30 min, then incubated with rabbit anti-CDK5 antibody (Santa Cruz, Cat. No. sc-173, 1:50 diluted) in PBT1 overnight at 4°C. Afterward, samples were incubated with Alexa Fluor^®^ 488 donkey anti-rabbit secondary antibody (Invitrogen, Cat. No. A21206, 1:200 diluted) in PBT2 for 1 h, followed by incubation with DAPI (Gene View Scientific Inc.) in PBS for 1 h. Lastly, samples were mounted in PBS/glycerol (1:1) and imaged with a confocal microscope (LSM 700, Zeiss, Germany).

### Auditory Brainstem Responses (ABR) Measurement

Mice were placed on an isothermal pad to keep the body temperature at 37°C during the whole experiment. A RZ6 workstation and BioSig software (Tucker Davis Technologies) were used for stimulus generation, presentation, ABR acquisition, and data management. After the mice were anesthetized by intraperitoneally injecting 5% chloral hydrate for 0.5 ml/100 g body weight, electrodes were inserted subcutaneously at the vertex and pinna as well as near the tail. Acoustic stimuli (clicks or pure-tone bursts) of decreasing sound level from 90 dB SPL in 10 dB SPL steps were generated using high-frequency transducers. At each sound level, 512 responses were sampled and averaged. Hearing threshold of each mouse was determined as the lowest sound level at which all ABR waves were detectable.

### Distortion Product Otoacoustic Emission (DPOAE) Measurement

Mice were anesthetized and maintained as described above. Two sine-wave tones of different frequencies (F1 and F2, F2 = 1.2 × F1) were presented for 1-s durations ranging from 20 to 80 dB SPL in 10 dB SPL steps using two speakers (Tucker-Davis Technologies, MF1, upper frequency limit 88 kHz). The emitted acoustic signal was picked up by a microphone (Etymotic Research, ER10B+, upper frequency limit 32 kHz) and digitized, from which the magnitude of the distortion product (frequency = 2 × F1 – F2) was determined. The surrounding noise floor was calculated by averaging adjacent frequency bins near the distortion product frequency. DPOAE thresholds were calculated when the magnitude of the distortion product was at least 5 dB SPL higher than the noise floor.

### Scanning Electron Microscopy (SEM)

Mouse inner ears were fixed with 2.5% glutaraldehyde in 0.1 M phosphate buffer overnight at 4°C. Cochleae were dissected out of the temporal bone, then post-fixed with 1% osmium tetroxide in 0.1 M phosphate buffer at 4°C for 2 h. After dehydration in ethanol, samples were critically point dried using a Leica EM CPD300 (Leica, Germany), then mounted and sputter coated with platinum (15 nm) using a Cressington 108 sputter coater (Cressington, United Kingdom). Images were taken using a Quanta250 field-emission scanning electron microscope (FEI, Netherlands).

### Western Blot

Mouse tissues were homogenized in ice-cold lysis buffer consisting of 150 mM NaCl, 50 mM Tris at pH 7.5, 1% NP-40, 0.1% SDS, 1% (vol/vol) Triton X-100, 1 mM PMSF, and 1 × protease inhibitor cocktail (Sigma-Aldrich, Saint Louis, MO, United States). The supernatant was collected after centrifugation at 4°C and separated by polyacrylamide gel electrophoresis (PAGE), then transferred to PVDF membrane. After blocking in PBS containing 5% milk and 0.1% Tween-20, the membrane was incubated with primary antibody at 4°C over night, followed by incubation with HRP-conjugated secondary antibody (Bio-Rad, Cat. No. 170-6515 or 170-6516) at room temperature for an hour. The signals were detected with the ECL system (Cell Signaling Technology, Danvers, MA, United States). Primary antibodies used are as follows: anti-ERM (rabbit, Cell Signaling Technology, Cat. No. 3142), anti-pERM (rabbit, Cell Signaling Technology, Cat. No. 3726), anti-CDK5 (rabbit, Santa Cruz, Cat. No. sc-173), and anti-GAPDH (mouse, Millipore, Cat. No. MAB374).

### FM 1-43FX Uptake Assay

FM1-43FX (Molecular Probes, Invitrogen), a fixable analog of FM1-43 [*N*-(3-triethylammoniumpropyl)-4-(4-(dibutylamino)-styryl) pyridinium dibromide] was used to label functional hair cells. Briefly, dissected mouse basilar membrane was incubated with 3 μM FM1-43FX in PBS for 30 s and rinsed three times in PBS, then fixed with 4% PFA at room temperature for 20 min. The samples were mounted in PBS-glycerol (1:1) and imaged with an epifluorescence microscope (IX53, Olympus, Japan).

### Statistical Analysis

Data were shown as means ± standard error of mean. One way ANOVA with Dunnett’s test was used to determine statistical significance, and *p* < 0.05 was considered statistically significant.

## Results

### CDK5 Is Expressed in Mouse Cochlear Hair Cells

**FIGURE 1:**
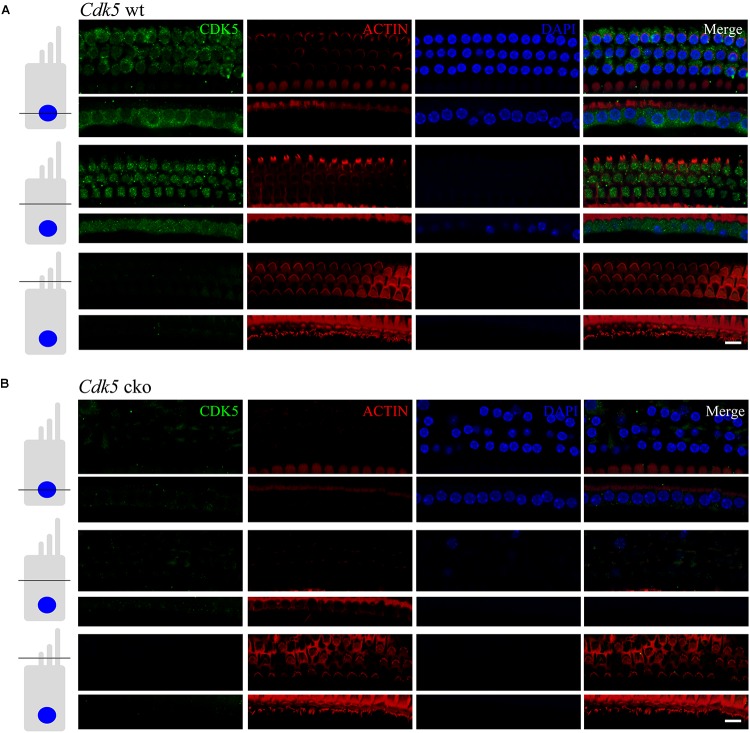
CDK5 is expressed in the cell body of mouse auditory hair cells. Expression of CDK5 in auditory hair cells was examined by performing whole-mount immunostaining of *Cdk5^lox/lox^* **(A)** and *Atoh1^Cre/+^*;*Cdk5^lox/lox^* **(B)** cochlea. F-actin core of stereocilia was visualized by TRITC-conjugated phalloidin. Nuclei were visualized by DAPI. Images were taken from the middle turn of 5-month-old mouse cochlea using a confocal microscope at different levels as indicated. Scale bar, 10 μm.

The expression of CDK5 in mouse cochlea was examined by performing whole-mount immunostaining using a specific anti-CDK5 antibody. The results showed that CDK5 immunoreactivity was present in the cell body of both IHCs and OHCs, but not in the stereocilia (Figure 1A). Cryosection immunostaining confirmed that CDK5 was present in the cell body of both IHCs and OHCs, and the immunoreactivity was more concentrated at the apical surface of hair cells (Supplementary Figure S1A).

In order to examine the physiological role of CDK5, we crossed *Cdk5^lox/+^* mice with *EIIa^Cre/+^* mice to generate *EIIa^Cre/+^*;*Cdk5^lox/+^* mice, which were then crossed with *Cdk5^lox/+^* mice to obtain *EIIa^Cre/+^*;*Cdk5^lox/lox^* mice. *Cdk5* expression is disrupted in all tissues in *EIIa^Cre/+^*;*Cdk5^lox/lox^* mice, hence we designate them as “*Cdk5* ko mice” in the following text. RT-PCR and western blot confirmed that CDK5 expression is indeed abolished in various tissues of *Cdk5* ko mice (data not shown). *Cdk5* ko mice died perinatally as reported (Ohshima et al., 1996), preventing us from further analyzing the effect of *Cdk5* inactivation on hearing. To disrupt *Cdk5* expression specifically in the hair cells, we utilized *Atoh1^Cre/+^* mice that express Cre recombinase in the developing cochlear hair cells from approximately E14.5 (Yang et al., 2010). We designate the obtained *Atoh1^Cre/+^*;*Cdk5^lox/lox^* mice as “*Cdk5* cko mice” in the following text. Whole-mount and cryosection immunostaining confirmed that CDK5 is absent in the cochlear hair cells of *Cdk5* cko mice (Figure 1B and Supplementary Figure S1B). In the following experiments, *Cdk5^lox/lox^* mice (“*Cdk5* wt mice”) were used as control.

### *Cdk5* Inactivation in Hair Cells Causes Hearing Loss

**FIGURE 2:**
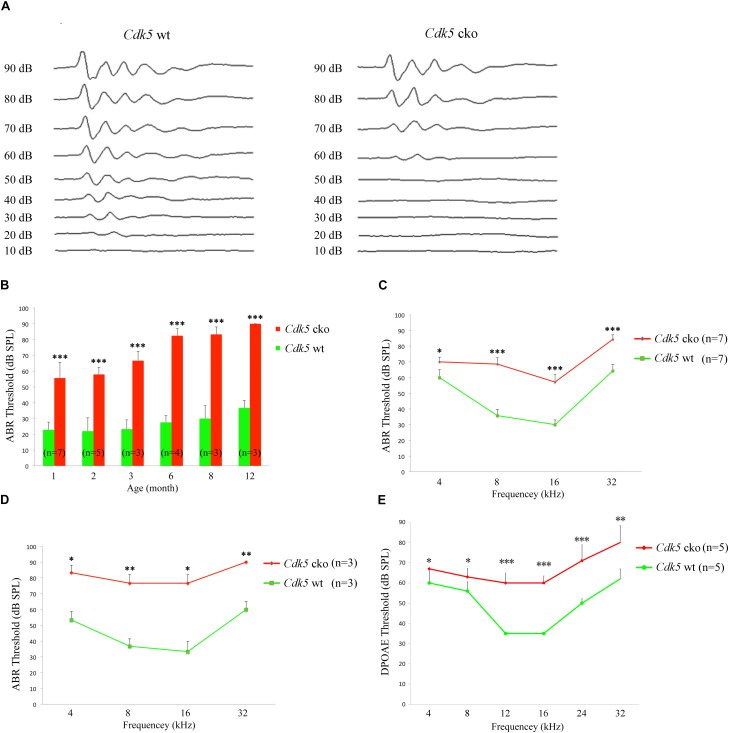
Hearing threshold is significantly elevated in *Cdk5* cko mice. **(A)** Raw traces of ABR responses to click stimuli in 1-month-old *Atoh1^Cre/+^*;*Cdk5^lox/lox^* and *Cdk5^lox/lox^* mice. **(B)** The ABR thresholds for click stimuli in *Atoh1^Cre/+^*;*Cdk5^lox/lox^* and *Cdk5^lox/lox^* mice at different ages. **(C)** The ABR thresholds for pure tone stimuli in 1-month-old *Atoh1^Cre/+^*;*Cdk5^lox/lox^* and *Cdk5^lox/lox^* mice. **(D)** The ABR thresholds for pure tone stimuli in 8-month-old *Atoh1^Cre/+^*;*Cdk5^lox/lox^* and *Cdk5^lox/lox^* mice. **(E)** The DPOAE thresholds for pure tone stimuli in 1-month-old *Atoh1^Cre/+^*;*Cdk5^lox/lox^* and *Cdk5^lox/lox^* mice. The numbers of animals for each group used in the experiments are indicated. ^∗^*p* < 0.05; ^∗∗^*p* < 0.01; ^∗∗∗^*p* < 0.001.

The auditory function of *Cdk5* cko mice was evaluated by performing auditory brainstem response (ABR) measurement, which detects the sound-evoked electrophysiological potentials in the auditory pathway from the cochlea to the brainstem. The results revealed significant differences in click-induced ABR thresholds between *Cdk5* cko mice and control mice. The ABR threshold of 1-month-old *Cdk5* cko mice was about 30 dB higher than that of control mice (Figures 2A,B). The ABR threshold elevation of *Cdk5* cko mice increased to about 40 dB at age of 3 months, and reached around 50 dB after age of 6 months (Figure 2B). Elevated ABR thresholds were also observed in *Cdk5* cko mice to pure tone stimuli of all examined frequencies in an age-dependent manner (Figures 2C,D).

Distortion product otoacoustic emission (DPOAE) measurements were then performed to examine the function of OHCs in *Cdk5* cko mice. DPOAE detects a low-level sound that is generated by functional OHCs and emitted back to the ear canal. The results showed that the DPOAE threshold of *Cdk5* cko mice was significantly elevated compared to that of control mice, suggesting that there were OHC function deficits in *Cdk5* cko mice (Figure 2E). Taken together, our present data suggested that *Cdk5* inactivation in hair cells compromises OHC function and causes hearing loss.

### *Cdk5* Inactivation in Hair Cells Does Not Affect Stereocilia Development and MET

**FIGURE 3:**
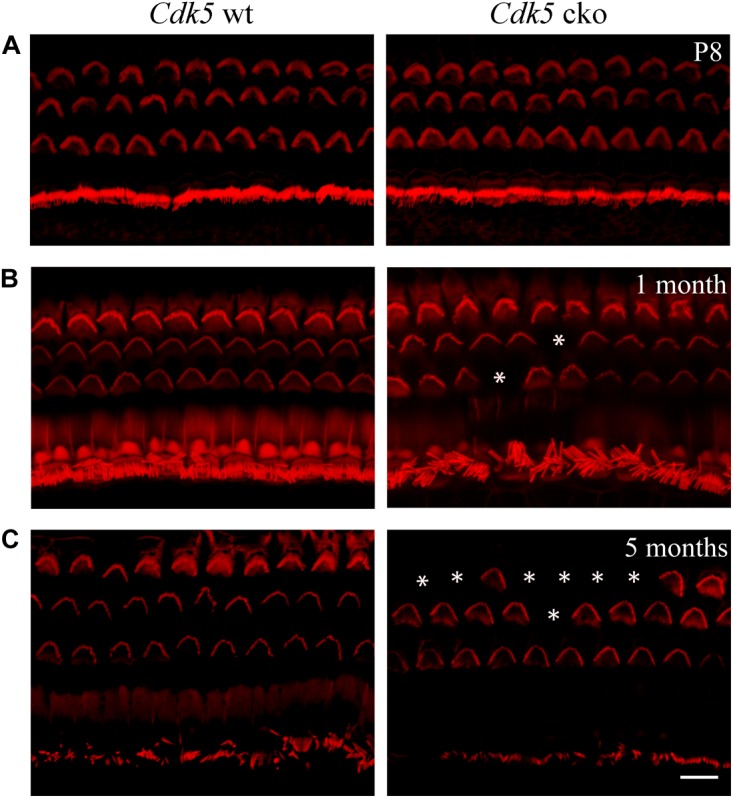
Stereocilia morphology examined by phalloidin staining at different developmental stages. Phalloidin staining and confocal microscopy were employed to examine the morphology of P8 **(A)**, 1-month-old **(B)**, and 5-month-old **(C)** *Atoh1^Cre/+^*; *Cdk5^lox/lox^* and *Cdk5^lox/lox^* hair cell stereocilia at the middle turn of cochleae. Loss of stereocilia was indicated by asterisks. Scale bar, 10 μm.

**FIGURE 4:**
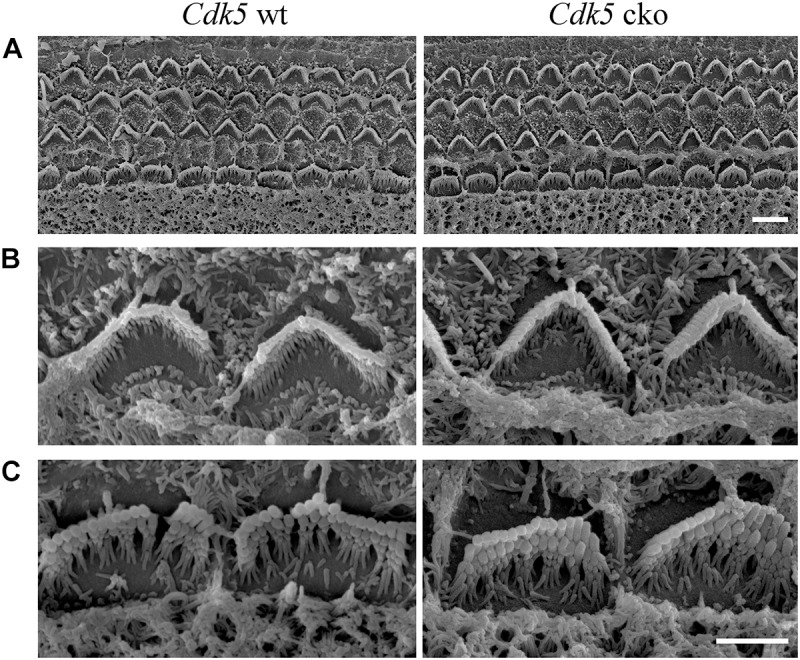
Hair bundle morphology of P8 *Cdk5* cko mice examined by SEM. **(A)** Low magnification images of hair bundles at the middle turn of *Cdk5^lox/lox^* and *Atoh1^Cre/+^*; *Cdk5^lox/lox^* cochleae. Scale bar, 5 μm. **(B)** High magnification images of hair bundles of *Cdk5^lox/lox^* and *Atoh1^Cre/+^*; *Cdk5^lox/lox^* OHCs. **(C)** High magnification images of hair bundles of *Cdk5^lox/lox^* and *Atoh1^Cre/+^*; *Cdk5^lox/lox^* IHCs. Scale bar, 2.5 μm.

The morphology of auditory hair cell stereocilia was examined by performing phalloidin staining of stereocilia’s F-actin core. At postnatal day 8 (P8), the morphology of cochlear hair cell stereocilia in *Cdk5* cko mice was largely unaffected (Figure 3A). Stereocilia morphology was further examined by performing SEM. Consistent with the phalloidin staining result, SEM showed that the stereocilia morphology of P8 *Cdk5* cko OHCs and IHCs was largely unaffected, forming regular staircase pattern and normal PCP (Figures 4A–C). Taken together, these results suggested that stereocilia development was not affected by *Cdk5* inactivation in hair cells.

Normal stereocilia development in *Cdk5* cko hair cells prompted us to examine whether the *Cdk5* cko hair cells are functionally intact. Florescent dye FM1-43 has been shown to enter hair cells through the MET channels, providing an indicator of the functional integrity of hair cells (Gale et al., 2001; Meyers et al., 2003). Here we used FM1-43FX, a fixable analog of FM1-43, to examine the function integrity of *Cdk5* cko hair cells. The results showed that FM1-43FX uptake of P8 *Cdk5* cko hair cells was indistinguishable from that of control hair cells, suggesting that hair cell function is not affected in newborn *Cdk5* cko mice (Supplementary Figure S2).

### *Cdk5* Inactivation in Hair Cells Affects Stereocilia Maintenance and Causes Hair Cell Loss

**FIGURE 5:**
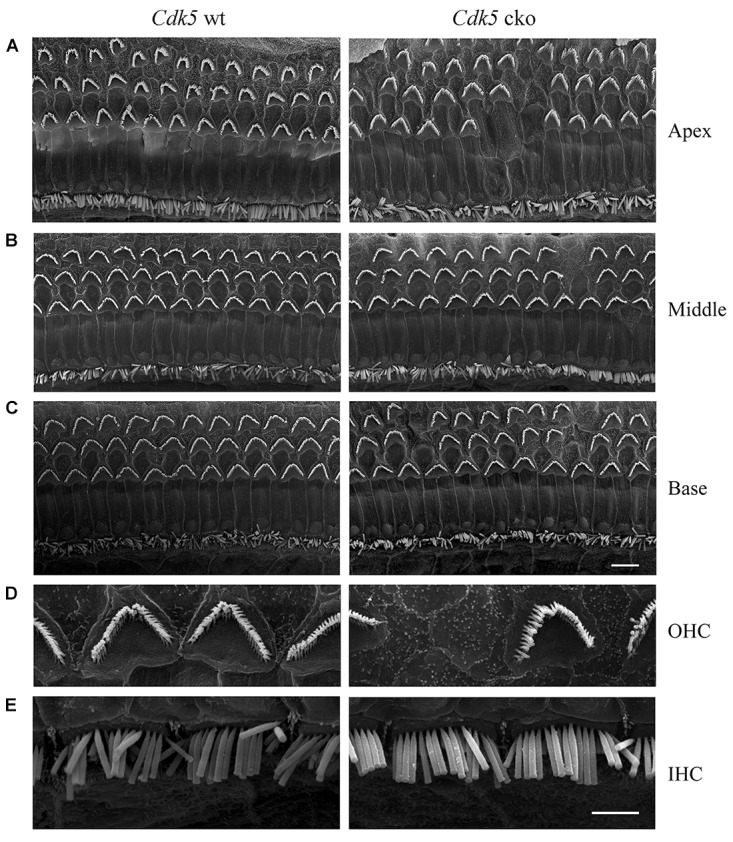
Hair bundle morphology of 1-month-old *Cdk5* cko mice examined by SEM. **(A)** Low magnification images of hair bundles of *Cdk5^lox/lox^* and *Atoh1^Cre/+^*; *Cdk5^lox/lox^* cochleae at apical turn. **(B)** Low magnification images of hair bundles of *Cdk5^lox/lox^* and *Atoh1^Cre/+^*; *Cdk5^lox/lox^* cochleae at middle turn. **(C)** Low magnification images of hair bundles of *Cdk5^lox/lox^* and *Atoh1^Cre/+^*; *Cdk5^lox/lox^* cochleae at basal turn. Scale bar, 5 μm. **(D)** High magnification images of hair bundles of middle turn *Cdk5^lox/lox^* and *Atoh1^Cre/+^*; *Cdk5^lox/lox^* OHCs. **(E)** High magnification images of hair bundles of middle turn *Cdk5^lox/lox^* and *Atoh1^Cre/+^*; *Cdk5^lox/lox^* IHCs. Scale bar, 2 μm.

**FIGURE 6:**
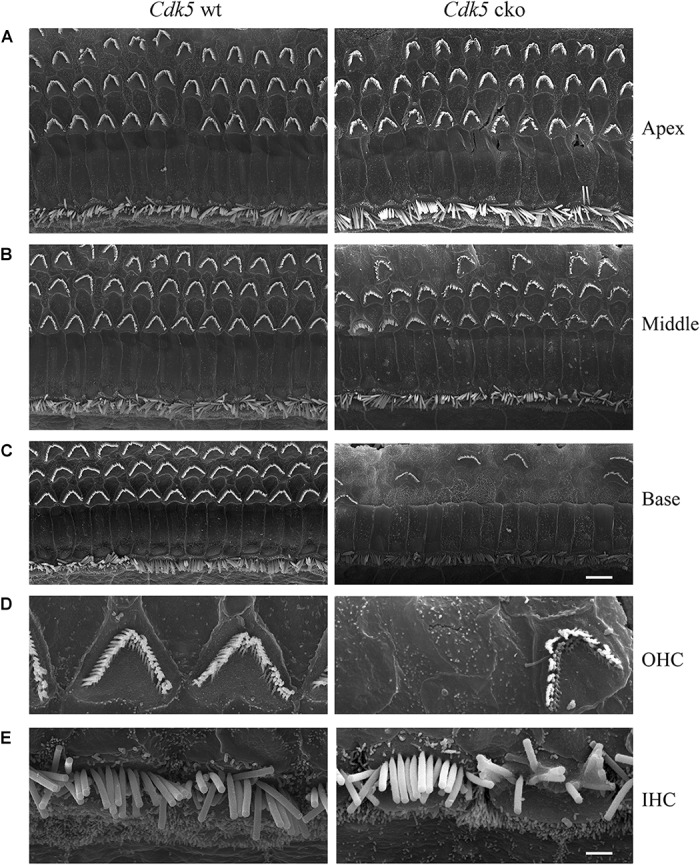
Hair bundle morphology of 5-month-old *Cdk5* cko mice examined by SEM. **(A)** Low magnification images of hair bundles of *Cdk5^lox/lox^* and *Atoh1^Cre/+^*; *Cdk5^lox/lox^* cochleae at apical turn. **(B)** Low magnification images of hair bundles of *Cdk5^lox/lox^* and *Atoh1^Cre/+^*; *Cdk5^lox/lox^* cochleae at middle turn. **(C)** Low magnification images of hair bundles of *Cdk5^lox/lox^* and *Atoh1^Cre/+^*;*Cdk5^lox/lox^* cochleae at basal turn. Scale bar, 5 μm. **(D)** High magnification images of hair bundles of middle turn *Cdk5^lox/lox^* and *Atoh1^Cre/+^*; *Cdk5^lox/lox^* OHCs. **(E)** High magnification images of hair bundles of middle turn *Cdk5^lox/lox^* and *Atoh1^Cre/+^*; *Cdk5^lox/lox^* IHCs. Scale bar, 1 μm.

In contrast to the normal stereocilia morphology observed at P8, phalloidin staining revealed that stereocilia degeneration took place in some of the *Cdk5* cko OHCs at age of 1-month, which was exacerbated furthermore at age of 5-months (Figures 3B,C). SEM confirmed the phalloidin staining results. By age of 1-month, complete stereocilia degeneration could be observed in some *Cdk5* cko OHCs (Figures 5A–E). By age of 5-months, more OHCs of *Cdk5* cko mice showed stereocilia degeneration, especially at the basal turn (Figures 6A–D). Meanwhile, stereocilia fusion could be observed in some IHCs of 5-month-old *Cdk5* cko mice (Figure 6E). Taken together, our data suggested that although *Cdk5* inactivation does not affect stereocilia development, it causes serious stereocilia maintenance deficits.

**FIGURE 7:**
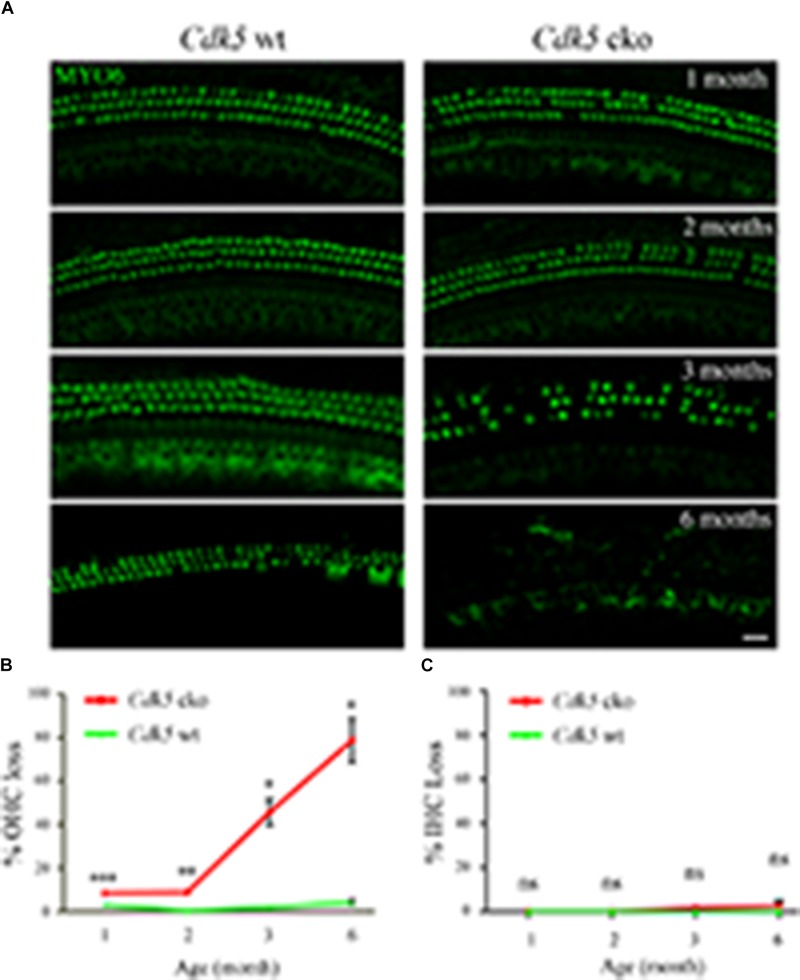
OHC loss was increased in *Cdk5* cko mice. **(A)** Whole-mount immunostaining was performed using an anti-MYO6 antibody at different ages as indicated. Shown are single confocal images of basal-middle turn cochlea. Scale bar, 20 μm. Please notice that MYO6 immunoreactivity in most IHCs is rather weak because they are not at the same focus plane as the OHCs. **(B)** OHC loss was quantified according to the results from **(A)**. **(C)** IHC loss was quantified according to the results from **(A)**. Numbers of mice used in each group are more than three. ^∗^*p* < 0.05; ^∗∗^*p* < 0.01; ^∗∗∗^*p* < 0.001; ns, not significant.

Stereocilia degeneration is usually followed by hair cell loss, hence we examined hair cells by performing whole-mount immunostaining using an antibody that detects hair cell marker myosin VI (MYO6) (Torii et al., 2016; Wong et al., 2016). The results showed that compared to control mice, there was increased OHC loss in *Cdk5* cko mice, which is exacerbated when mice age (Figure 7A). At age of 1-month, less than 10% of OHC was lost at the basal-middle turn cochlea of *Cdk5* cko mice, and this number reached around 80% by age of 6-months (Figure 7B). In contrast, IHC loss in *Cdk5* cko mice was negligible (Figures 7A,C).

### Ribbon Synapse Formation Is Unaffected in *Cdk5* cko Mice

**FIGURE 8:**
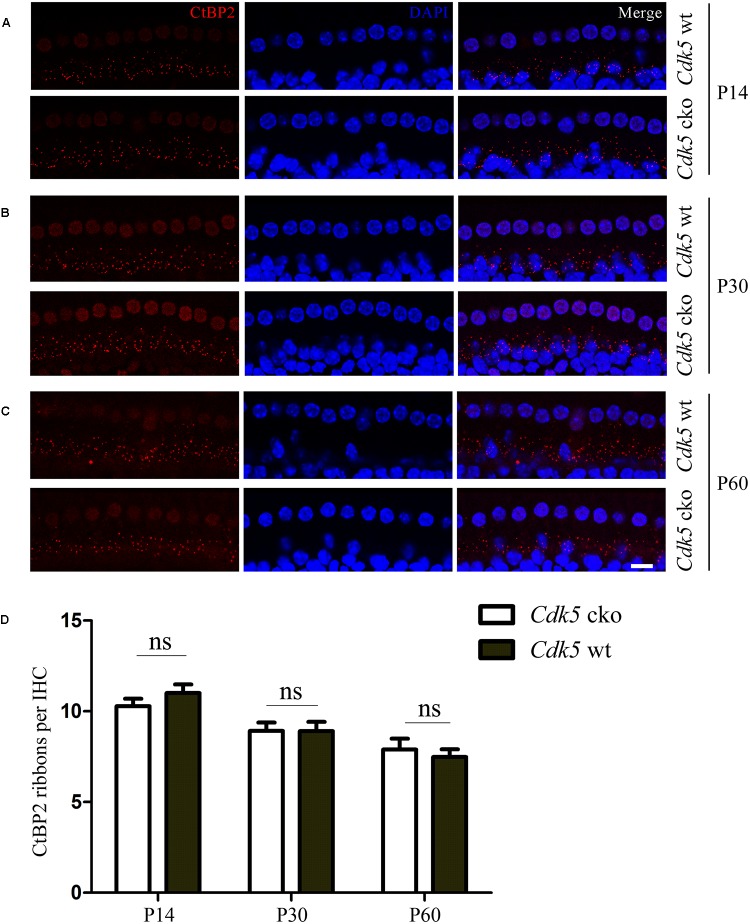
Ribbon synapses are unaffected in *Cdk5* cko mice. **(A)** Whole-mount immunostaining of CtBP2 in P14 *Atoh1^Cre/+^*; *Cdk5^lox/lox^* and *Cdk5^lox/lox^* IHCs. **(B)** Whole-mount immunostaining of CtBP2 in P30 *Atoh1^Cre/+^*; *Cdk5^lox/lox^* and *Cdk5^lox/lox^* IHCs. **(C)** Whole-mount immunostaining of CtBP2 in P60 *Atoh1^Cre/+^*; *Cdk5^lox/lox^* and *Cdk5^lox/lox^* IHCs. Nuclei were visualized by DAPI. Images were taken from the middle turn of cochleae using a confocal microscope. Scale bar, 10 μm. **(D)** CtBP2-positive ribbon synapses were quantified according to the results from **(A–C)**.

CDK5 has been shown to play important roles in synapse development and function (Tan et al., 2003; Fu et al., 2005; Lin et al., 2005; Lai and Ip, 2015). IHCs contain specialized synapses named ribbon synapses, which are also present in retinal photoreceptors, etc (Matthews and Fuchs, 2010). Ribeye/CtBP2 is one of the most abundant proteins in ribbon synapses and is commonly used as a marker for ribbon synapses (Schmitz et al., 2000). We then examined the integrity of hair cell ribbon synapses by performing whole-mount immunostaining with an anti-CtBP2 antibody. The results revealed no difference between *Cdk5* cko mice and control mice at different developmental stages (Figures 8A–D), suggesting that CDK5 inactivation does not affect the formation of ribbon synapses of cochlear hair cells.

### ERM Phosphorylation Is Decreased in the Inner Ear of *Cdk5* cko Mice

**FIGURE 9:**
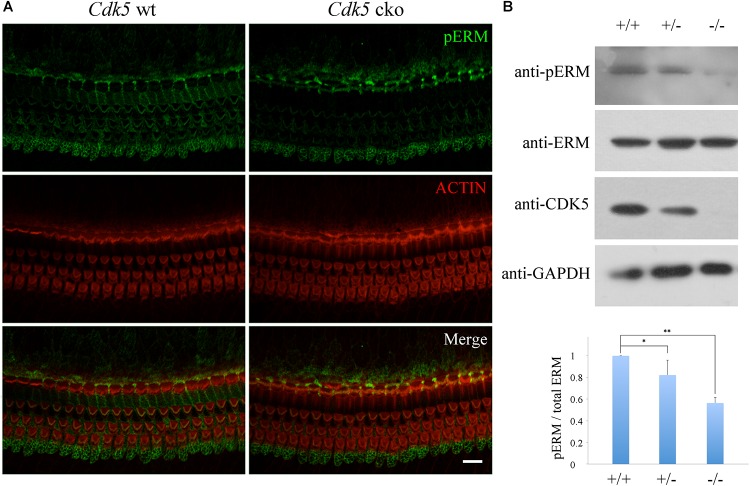
ERM phosphorylation is reduced by *Cdk5* inactivation. **(A)** Whole-mount immunostaining of phosphorylated ERM (pERM) in P8 *Atoh1^Cre/+^*; *Cdk5^lox/lox^* and *Cdk5^lox/lox^* organ of Corti. F-actin core of stereocilia was visualized by TRITC-conjugated phalloidin. Images were taken from the middle turn of cochleae using a confocal microscope. Scale bar, 10 μm. **(B)** Expression level of pERM, ERM, and CDK5 in the inner ear of E18.5 *Cdk5^+/+^*, *Cdk5^+/-^*, and *Cdk5^-/-^* mice was examined by western blot. GAPDH was included as internal control. The bar graph shows quantification of three independent experiments. ^∗^*p* < 0.05; ^∗∗^*p* < 0.01.

CDK5 phosphorylates many proteins that are involved in F-actin assembly and maintenance. ERM (ezrin, radixin, and moesin) proteins are known CDK5 substrates (Yang et al., 2003; Yang and Hinds, 2003), and have been shown to play important roles in stereocilia development and/or maintenance (Kitajiri et al., 2004; Pataky et al., 2004; Han et al., 2015). Hence we examined the effect of *Cdk5* inactivation on the phosphorylation status of ERM proteins in the cochleae. Whole-mount immunostaining showed that phosphorylated ERM (pERM) was readily detected in the stereocilia of control mice, but significantly decreased in the stereocilia of newborn *Cdk5* cko mice (Figure 9A and Supplementary Figure S3). Furthermore, western blot results confirmed that pERM was significantly reduced in the *Cdk5* ko inner ear at E18.5 (Figure 9B). Taken together, our data suggested that pERM is decreased in the stereocilia of *Cdk5* cko mice, which might contribute to the abnormal stereocilia maintenance.

## Discussion

CDK5 plays important roles in neural development and function, and dysfunction of CDK5 is associated with a number of neurological disorders (Su and Tsai, 2011). The role of CDK5 in the central nervous system has been extensively studied, whereas its role in peripheral sensory systems is less understood. It was shown that CDK5 and its activator p35 are highly expressed in primary afferent nociceptive fibers in mouse dorsal root ganglia, where they modulate nociceptive signaling through phosphorylation of transient receptor potential vanilloid 1 (TRPV1) (Pareek et al., 2006, 2007; Yang et al., 2007). CDK5 has also been shown to express in chicken auditory hair cells and regulate the membrane expression and kinetics of BK channel Slo through phosphorylation (Bai et al., 2012), but its physiological role in hearing has not been investigated.

In the present work, we showed that CDK5 was expressed in the cell body of mouse cochlear hair cells. The expression of *Cdk5* in mouse cochlear hair cells was supported by RNA transcriptome sequencing result (SHIELD^1^) (Shen et al., 2015). We found that CDK5 inactivation in hair cells led to severe stereocilia degeneration. Stereocilia are F-actin-based, microvilli-like protrusions on the apical surface of hair cells, and are indispensable for hearing transduction (Schwander et al., 2010). Stereocilia degeneration was mainly detected in the OHCs of *Cdk5* cko mice, while OHCs are known to play important roles in amplifying sound-evoked vibrations. Consequently, the observed OHC stereocilia degeneration might explain the profound hearing loss in *Cdk5* cko mice. Meanwhile, stereocilia disorganization was also observed in some IHCs, which might also contribute to the hearing loss in *Cdk5* cko mice.

Further investigation showed that phosphorylation of CDK5 substrate ERM proteins was significantly decreased in *Cdk5* cko hair cells, which might contribute to the eventual stereocilia degeneration. ERM proteins are evolutionary conserved group of three related proteins including ezrin, radixin, and moesin. ERM proteins crosslink actin filaments with plasma membrane, and play important roles in the organization of microvilli (Fehon et al., 2010). In the mouse cochlear hair cells, ezrin is expressed in the stereocilia at very low level, whereas moesin is not detected at all (Kitajiri et al., 2004). In contrast, radixin is intensely expressed in the stereocilia (Kitajiri et al., 2004). Mutations in the human *RDX* gene that encodes radixin are associated with non-syndromic hearing loss DFNB24 (Khan et al., 2007). In mice, *Rdx* deficiency causes progressive degeneration of cochlear stereocilia that eventually leads to hearing loss (Kitajiri et al., 2004). Similar phenotypes of stereocilia degeneration in *Rdx* ko mice and *Cdk5* cko mice are consistent with the hypothesis that CDK5 regulates stereocilia maintenance through phosphorylation of ERM proteins.

CDK5 has been suggested to play pivotal roles in synapse development and/or function (Lai and Ip, 2015). For example, it was shown that phosphorylation of liprinα1 by CDK5 mediates neuronal activity-dependent synapse development in the brain (Huang et al., 2017). *Cdk5* inactivation causes morphological abnormalities at the neuromuscular junction (NMJ) both pre- and post-synaptically (Fu et al., 2005). However, our present data showed that CtBP2 immunoreactivity in *Cdk5* cko hair cells was comparable to that in control mice, suggesting that *Cdk5* inactivation does not affect the development of hair cell ribbon synapses. Whether the function of hair cell ribbon synapses is affected by *Cdk5* inactivation is not examined in the present study and awaits further investigation.

Besides stereocilia degeneration, robust OHC loss was also observed in *Cdk5* cko mice. Our present hypothesis is that *Cdk5* inactivation causes stereocilia degeneration through dysregulated ERM phosphorylation, which then leads to hair cell degeneration. However, possibility remains that stereocilia degeneration and hair cell degeneration might happen independently to each other in *Cdk5* cko mice, or alternatively, stereocilia degeneration is even a result of hair cell degeneration. It has been reported that CDK5 could directly regulate cell survival, and that CDK5 dysfunction could lead to cell death (Zhou et al., 2015; Shi et al., 2016; Nikhil and Shah, 2017; NavaneethaKrishnan et al., 2018). Identification of hair cell-expressed CDK5 substrates other than ERM and Slo will help us to address this question.

A pharmacological inhibitor of CDK5, roscovitine, has been shown to significantly increase the number of hair cells and supporting cells in rat cochlear cultures through triggering differentiation of precursor cells (Malgrange et al., 2003). In the present work, however, supernumerary hair cells and supporting cells were not observed in *Cdk5* cko cochleae. This discrepancy might result from the fact that CDK5 was inactivated only in hair cells of *Cdk5* cko mice in the present work, whereas roscovitine affected both hair cell and supporting cells in the cochlear cultures. Additionally, it remains possible that roscovitine might trigger differentiation of cochlear precursor cells through inhibition of kinases other than CDK5. In line with this, roscovitine has been shown to also inhibit CDK1and CDK2, though with a lower efficiency (Meijer et al., 1997), and expression of CDK1 and CDK2 has been detected in rat hair cells and/or supporting cells (Malgrange et al., 2003).

As mentioned above, the kinase activity of CDK5 needs to be activated by specific activators. Three CDK5 activators have been identified, namely p35, p39, and CCNI (Tsai et al., 1994; Tang et al., 1995; Brinkkoetter et al., 2009). Inactivation of CDK5 activator(s) has been shown to cause neuronal deficits (Su and Tsai, 2011; Luo et al., 2016; Kamiki et al., 2018). Recently, our group reported a novel CDK5 activator, CCNI2, which is present in human, chicken, and zebrafish, but not in mouse and rat (Liu et al., 2017). The expression of CDK5 activators in the inner ear has not been reported. However, RNA transcriptome sequencing revealed that *p35* and *Ccni*, but not *p39* transcript, were expressed in mouse cochlear hair cells (Shen et al., 2015). At present, whether p35 or CCNI is necessary for CDK5 activation in hair cells remains unknown and awaits further investigation.

## Ethics Statement

All animal experimental procedures were approved by the Animal Ethics Committee of Shandong University School of Life Sciences (Permit Number: SYDWLL-2017-05) and performed accordingly.

## Author Contributions

ZX conceived the study. XZ, CL, BZ, and YW performed the experiments. All authors analyzed the data. ZX wrote the manuscript.

## Conflict of Interest Statement

The authors declare that the research was conducted in the absence of any commercial or financial relationships that could be construed as a potential conflict of interest.

## Footnotes

1 https://shield.hms.harvard.edu
