# Supplementary material for: Inactivation of Cyclin-Dependent Kinase 5 in Hair Cells Causes Hearing Loss in Mice
Source: Front Mol Neurosci. 2018 Dec 11;11:461. doi: 10.3389/fnmol.2018.00461 (PMC6297389; doi:10.3389/fnmol.2018.00461)
Supplement: Supplementary file 1 [file Data_Sheet_1.docx]

**Inactivation of cyclin-dependent kinase 5 in hair cells causes hearing loss in mice**

Xiaoyan Zhai^1#^, Chengcheng Liu^1,2#^, Bin Zhao^1^, Yanfei Wang^1^, Zhigang Xu^1,3,4*^

^1^Shandong Provincial Key Laboratory of Animal Cells and Developmental Biology, School of Sciences, Shandong University, Qingdao, Shandong 266237, China

^2^Department of Otolaryngology-Head and Neck Surgery, The Second Hospital of Shandong University, Jinan 250033, China

^3^Shenzhen Research Institute of Shandong University, Shenzhen, Guangdong 518057, China

^4^Shandong Provincial Collaborative Innovation Center of Cell Biology, Shandong Normal University, Jinan, Shandong 250014, P. R. China

^#^Contributed equally to this work

^*^Corresponding author:

Zhigang Xu, E-mail: xuzg@sdu.edu.cn, Tel: 86-532-58630869


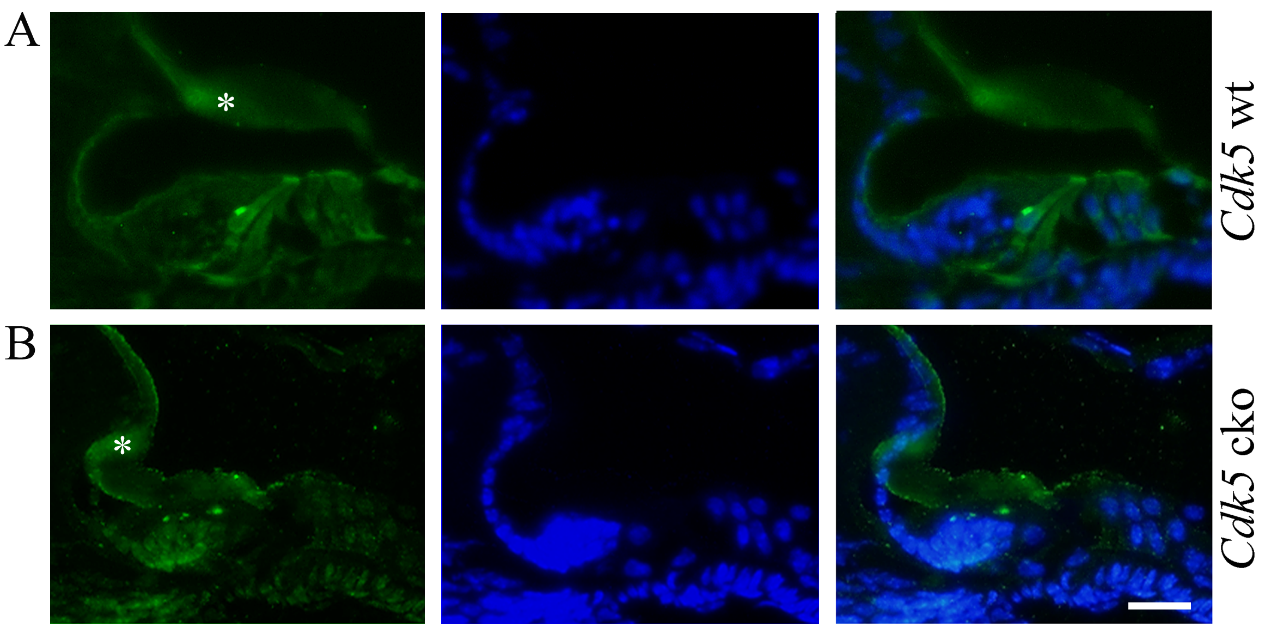


**Fig. S1**. **CDK5 is expressed in mouse auditory hair cells.** Cryosection immunostaining of CDK5 in *Cdk5^lox/lox^* (A) and *Atoh1^Cre/+^*;*Cdk5^lox/lox^* (B) cochleae at P8. Nuclei were visualized by DAPI. Images were taken from the middle turn of mouse cochlea using a confocal microscope. Asterisks indicate unspecific staining of tectorial membrane. Scale bar, 10 μm.

**
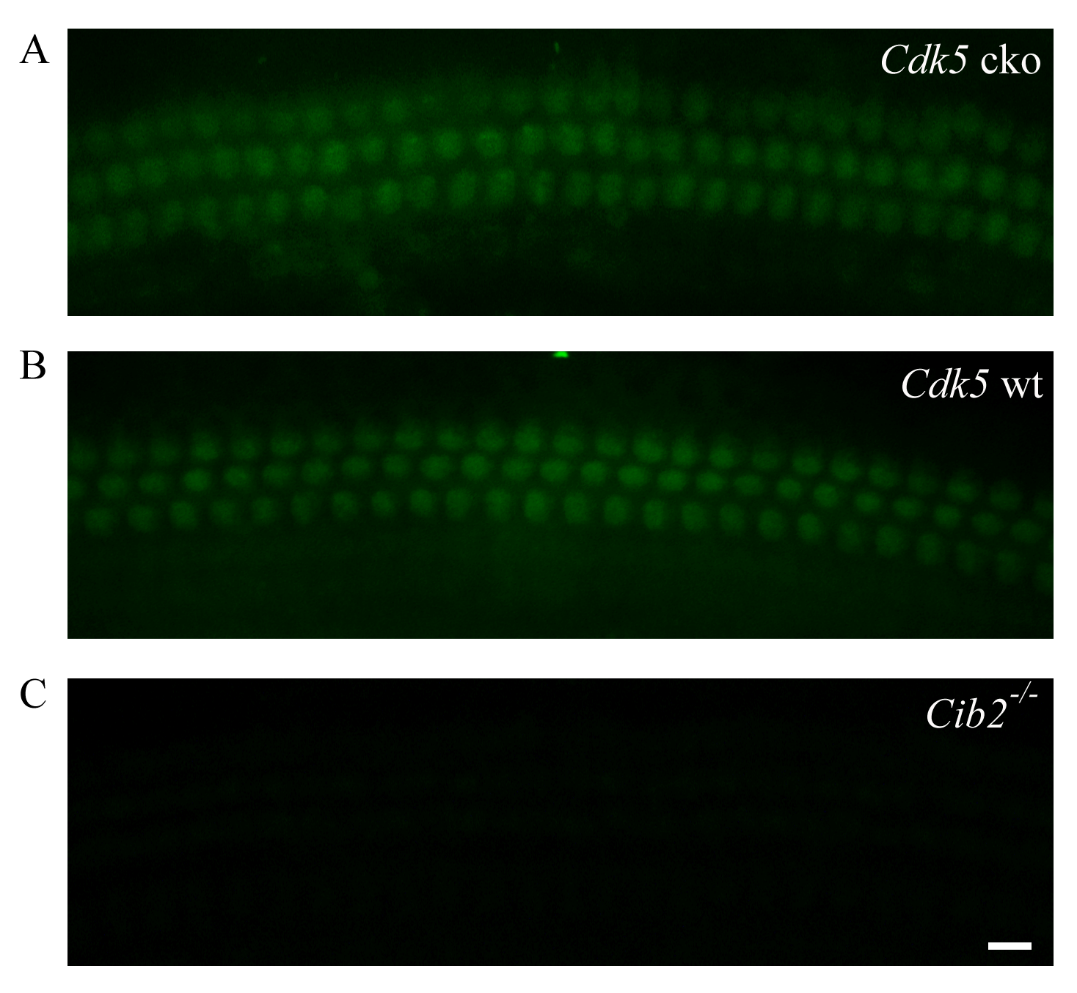
**

**Fig. S2. Hair cells of *Cdk5* cko mice are functionally normal.** FM1-43FX uptake by auditory hair cells of P8 *Atoh1^Cre/+^*;*Cdk5^lox/lox^* (A), *Cdk5^lox/lox^* (B), and *Cib2^-/-^* (C) mice were examined using an epifluorescence microscope. CIB2 is indispensable for mechanoelectrical transduction of hair cells, hence *Cib2^-/-^* mice was included in this experiment as negative control. Scale bar, 10 μm.

**
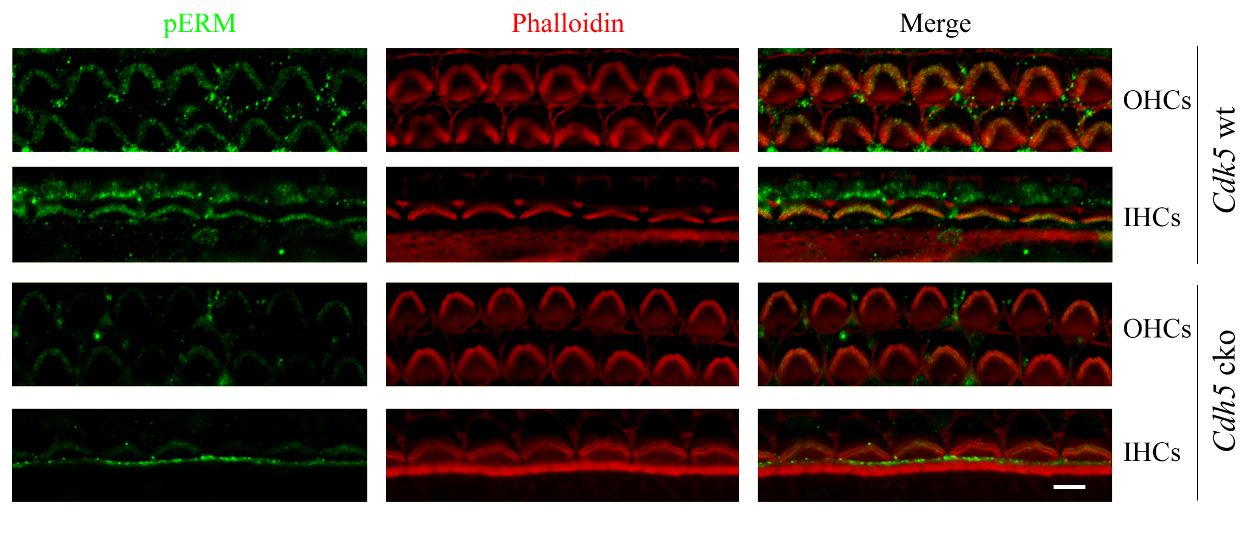
**

**Fig. S3. ERM phosphorylation is reduced by *Cdk5* inactivation.** Whole-mount immunostaining of phosphorylated ERM (pERM) in P4 *Atoh1^Cre/+^*;*Cdk5^lox/lox^* and *Cdk5^lox/lox^* organ of Corti. F-actin core of stereocilia was visualized by TRITC-conjugated phalloidin. Images were taken from the middle turn of cochleae using a confocal microscope. Scale bar, 5 μm.
